# The Practicability of a Novel Prognostic Index (PI) Model and Comparison with Nottingham Prognostic Index (NPI) in Stage I–III Breast Cancer Patients Undergoing Surgical Treatment

**DOI:** 10.1371/journal.pone.0143537

**Published:** 2015-11-23

**Authors:** Jiahuai Wen, Feng Ye, Shuaijie Li, Xiaojia Huang, Lu Yang, Xiangsheng Xiao, Xiaoming Xie

**Affiliations:** Department of Breast Oncology, Sun Yat-Sen University Cancer Center, State Key Laboratory of Oncology in South China, Collaborative Innovation Center for Cancer Medicine, Guangzhou, Guangdong, China; University of North Carolina School of Medicine, UNITED STATES

## Abstract

**Background:**

Previous studies have indicated the prognostic value of various laboratory parameters in cancer patients. This study was to establish a prognostic index (PI) model for breast cancer patients based on the potential prognostic factors.

**Methods:**

A retrospective study of 1661 breast cancer patients who underwent surgical treatment between January 2002 and December 2008 at Sun Yat-sen University Cancer Center was conducted. Multivariate analysis (Cox regression model) was performed to determine the independent prognostic factors and a prognostic index (PI) model was devised based on these factors. Survival analyses were used to estimate the prognostic value of PI, and the discriminatory ability of PI was compared with Nottingham Prognostic Index (NPI) by evaluating the area under the receiver operating characteristics curves (AUC).

**Results:**

The mean survival time of all participants was 123.6 months. The preoperative globulin >30.0g/L, triglyceride >1.10mmol/L and fibrinogen >2.83g/L were identified as risk factors for shorter cancer-specific survival. The novel prognostic index model was established and enrolled patients were classified as low- (1168 patients, 70.3%), moderate- (410 patients, 24.7%) and high-risk groups (83 patients, 5.0%), respectively. Compared with the low-risk group, higher risks of poor clinical outcome were indicated in the moderate-risk group [Hazard ratio (HR): 1.513, 95% confidence interval (CI): 1.169–1.959, *p* = 0.002] and high-risk group (HR: 2.481, 95%CI: 1.653–3.724, *p*< 0.001).

**Conclusions:**

The prognostic index based on three laboratory parameters was a novel and practicable prognostic tool. It may serve as complement to help predict postoperative survival in breast cancer patients.

## Introduction

Breast cancer is by far the most commonly diagnosed malignancy and second leading cause of cancer death in women globally. Estimated 232,670 new cases and 40,000 deaths occurred in the United States in 2014 according to American Cancer Society [1], which places heavy a burden on both patients and healthcare system.

Owing to the breakthrough in the treatments of breast cancer, including surgical resection, adjuvant chemotherapy, radiotherapy, endocrine therapy and targeted therapy, the overall survival improved and tumor recurrence reduced during the last decades [2]. However, recurrence and metastasis remain the main challenge for cure [3].

Up to now, useful prognostic factors can identify groups of patients with various prognoses. Clinical and pathological parameters have been used as prognostic factors to predict the outcome of breast cancer patients and help to develop appropriate adjuvant treatments for high-risk patients. The American Joint Committee on Cancer (AJCC) tumor-node-metastasis (TNM) staging system and the molecular subtypes classification are important prognostic predictors [4,5]. Clinically, the breast cancer is classified into several molecular subtypes based on the estrogen receptor (ER), progesterone receptor (PR), human epidermal growth factor receptor-2 (HER2) and Ki-67 index status. Patients with HER2 over-expressing subtype (ER-, PR- and HER2+) and triple-negative subtype (ER-, PR- and HER2-) suffer more recurrence, distant metastasis and drug resistance than the luminal subtype [6]. In addition to the tumor stage and molecular subtypes, various clinical tools were developed to predict the prognosis of breast cancer patients, such as 21-gene RT-PCR assay recurrence score, Nottingham Prognostic Index (NPI) and Adjuvant Online (Adjuvant!)[7]. The NPI combines three prognostic factors: nodal status, tumor size and histological grade [8]. Since the three components of the NPI formula reflect the metastatic potential and genetic instability of tumor cells, higher NPI generally indicates worse clinical outcomes. Patients were classified into four subsets with different probability of cancer-related death: excellent, good, moderate and poor groups [9].

Recent studies had investigated the association between laboratory parameters and the cancers. Being members of blood lipid profiles, total cholesterol level and triglyceride level were significantly higher in breast cancer patients [10,11]. Elevated cholesterol level and triglyceride level were risk factors for poor prognosis in lung cancer and prostate cancer [12,13]. In addition, previous studies demonstrated that fibrinogen participated in the tumorigenesis and progression [14,15] and elevated serum fibrinogen level predict poor clinical outcomes in several cancers [16,17,18].

In this study, we investigated the laboratory parameters routinely tested prior to the surgical treatment and set up a prognostic index model based on the independent prognostic factors. The prognostic model was assessed and compared with the NPI to estimate the clinical value in breast cancer patients.

## Patients and Methods

### Study population

We retrospectively reviewed the medical records of consecutive patients with primary breast cancer from January 1, 2002 to December 31, 2008 in Sun Yat-sen University Cancer Center (SYSUCC). Pathological diagnosis was carefully confirmed by pathologists. Other inclusion criteria included: (1) received surgical treatment; (2) female; (3) diagnosed as invasive ductal carcinoma or invasive lobular carcinoma. We excluded patients who (1) received neoadjuvant chemotherapy before surgery; (2) had surgical treatment before admission; (3) with previous or coexisting cancers other than breast cancer; (4) confirmed metastasis. All patients were followed up until December 31, 2014 or date of cancer-related deaths. Routine tests and telephone counseling were performed to understand the patients’ condition during follow-up time.

### Clinical data collection

Patient characteristics and clinicopathological factors like age, menopausal status, pathological diagnosis, tumor size, axillary lymph nodal status, histologic grade, hormonal receptor and HER2 status, date of last follow-up or cancer-related death were collected. Laboratory parameters investigated as potential prognostic factors in the current study included albumin, globulin, lactate dehydrogenase (LDH), total bilirubin (TB), uric acid, cholesterol, triglyceride and fibrinogen. The blood samples were collected and measured by the automatic biochemical analyzer in the central laboratory before surgery. The tumor stages were classified according to the AJCC TNM staging system (the 7th edition). The molecular subtypes were as follow: Luminal A (ER +, PR +, HER2 -and Ki-67≤ 14%), Luminal B (ER + and HER2 + or Ki-67> 14%), HER2 over-expressing (ER -, PR -, HER2 +) and triple-negativer breast cancer (ER -, PR -, HER2 -). HER2 positive was defined as “3+” in immunohistochemical test or “positive” in HER2 fluorescence in situ hybridization test. The NPI was calculated by the formula of lymph node stage (1–3) + histologic grade (1–3) + 0.2×tumor size (cm) and patients were stratified into four groups as excellent (≤ 2.40), good (2.41–3.40), moderate (3.41–5.40) and poor (>5.4)[8,9]

### Statistical analyses

The primary end point was cancer-specific survival (CSS) calculated from the date of diagnosis to the date of cancer-related deaths or the last follow-up. Means and standard deviations were calculated, and differences were identified by *t* test. Difference between categories were evaluated using chi-square test. Receiver operating characteristics (ROC) curve analysis were performed to assess the prognostic value of each laboratory parameter and to set up the optimal cut-off points for potential prognostic factors. Univariate analysis and multivariate analysis (Cox regression model) were used to confirm the independent prognostic variables associated with CSS. The prognostic index (PI) model was established based on independent variables and enrolled patients were stratified into low-, moderate- and high-risk groups. Kaplan—Meier method was performed for survival analysis and compared by log-rank test. Hazard ratios (HRs) and 95% confidence intervals (CIs) were calculated from the Cox regression model, and a two-tailed *p* value <0.05 was considered statistically significant. The comparison between PI and NPI was made using the method of DeLonget al [19]. All statistical analyses were performed using SPSS 19.0 (SPSS Inc., Chicago, IL, USA).

### Ethics Statement

The study protocol was approved the by independent ethical committee/institutional review board of Sun Yat-sen University Cancer Center (SYSUCC), and written informed consent about the scientific research was obtained from each participant prior to surgery. Patient records were anonymized and de-identified prior to analysis.

## Results

There were 1661 female patients with primary non-metastatic invasive breast cancer enrolled in this study. The mean survival time of all participants was 123.6 months and patient characteristics were shown in Table 1. The mean age was 42.2 years old (range 22–74 years), and 150 (9.0%) patients were under the age of 35. Invasive ductal carcinoma was the predominant type (97.2%). Tumor sizes of T1, T2 and T3 were observed in 698 (42.0%), 876 (52.7%) and 87 (5.2%) of the patients respectively, and 893 (53.8%) patients suffered regional lymph node metastasis. 129 (7.7%), 374 (22.5%), 836 (50.4%) and 322 (19.4%) were classified as excellent, good, moderate and poor NPI groups respectively. Luminal subtype comprised 77.4% of total participants, and 183 (11.0%) and 193 (11.6%) were HER2 over-expressing subtype and triple-negative subtype respectively. 89.3% (1484/1661) of enrolled participants received chemotherapies and all were performed within 45 days after the surgeries. The anthracycline and/or taxane-containing chemotherapy was the main option for adjuvant cytotoxic chemotherapy. Radiotherapies were given sequentially to the patients with more than 3 metastatic lymph nodes or tumor >50 mm in greatest dimension or breast conserving surgery. Patients with ER/PR positivity received endocrine therapies for more than 4 years. Target therapy was not performed due to the unavailability of trastuzumab at that time.

**Table 1:** Clinicopathological characteristics and laboratory parameters of patients.

Characteristic	All patients (n = 1661)	Low-risk group (n = 1168)	Moderate-risk group (n = 410)	High-risk group (n = 83)	*p* value
Age					<0.001
≤ 35	150(9.0)	126(84.0)	24(16.0)	0(0)	
> 35	1511(91.0)	1042(69.0)	386(25.5)	83(5.5)	
Menopause					<0.001
Yes	433(26.1)	264(61.0)	134(30.9)	35(8.1)	
No	1228(73.9)	904(73.6)	276(22.5)	48(3.9)	
Tumor type					0.481
IDC	1615(97.2)	1136(70.3)	400(24.8)	79(4.9)	
ILC	46(2.8)	32(69.6)	10(21.7)	4(8.7)	
Histologic grade					0.886
G1	562(33.8)	396(70.5)	135(24.0)	31(5.5)	
G2	664(40.0)	465(70.0)	165(24.8)	34(5.1)	
G3	435(26.2)	307(70.6)	110(25.3)	18(4.1)	
Tumor size					0.805
T1	698(42.0)	799(71.5)	163(23.4)	36(5.2)	
T2	876(52.7)	607(69.3)	227(25.9)	42(4.8)	
T3	87(5.2)	62(71.3)	20(23.0)	5(5.7)	
Lymph node status					0.127
N0	768(46.2)	545(71.0)	188(24.5)	35(4.6)	
N1	448(27.0)	327(73.0)	98(21.9)	23(5.1)	
N2	259(15.6)	179(69.1)	70(29.1)	10(3.9)	
N3	186(11.2)	117(32.9)	54(29.0)	15(8.1)	
ER					0.550
Positive	1047(63.0)	743(71.0)	256(24.5)	48(4.6)	
Negative	614(37.0)	425(69.2)	154(25.1)	35(5.7)	
PR					0.123
Positive	1162(70.0)	831(71.5)	280(24.1)	51(4.4)	
Negative	449(30.0)	337(67.5)	130(26.1)	32(6.4)	
HER-2					0.042
Positive	393(23.7)	889(70.1)	324(25.6)	55(4.3)	
Negative	1268(76.3)	279(71.0)	86(21.9)	28(7.1)	
NPI					0.652
Excellent group	129(7.7)	91(70.5)	32(24.8)	6(4.7)	
Good group	374(22.5)	269(71.9)	86(23.0)	19(5.1)	
Moderate group	836(50.4)	593(70.9)	199(23.8)	44(5.3)	
Poor group	322(19.4)	215(66.8)	93(28.9)	14(4.3)	
Cancer-specific survival					<0.001
Alive	1365(82.2)	992(72.7)	319(23.4)	54(4.0)	
Death	296(17.8)	176(59.5)	91(30.7)	29(9.8)	
Albumin level (g/L,mean±SD)	43.8±4.1				
Globulin level (g/L,mean±SD)	28.3±4.5				
LDH (U/L,mean±SD)	161.2±55.0				
TB (umol/L, mean+ SD)	13.5±9.3				
Uric acid (umol/L, mean±SD)	283.0±80.4				
Cholesterol (mmol/L,mean±SD)	5.09±1.05				
Triglyceride (mmol/L,mean±SD)	1.37±0.94				
Plasma fibrinogen(g/L, mean±SD)	2.95±0.71				

Abbreviation: SD *standard deviation*, IDC *Invasive ductal carcinoma*, ILC *Invasive lobula carcinomar*, ER *Estrogen receptor*,PR *Progesterone receptor*, HER2 *Human epidermal growth factor receptor-2*, LDH *lactate dehydrogenase*, TB *total bilirubin*

**Fig 1:**
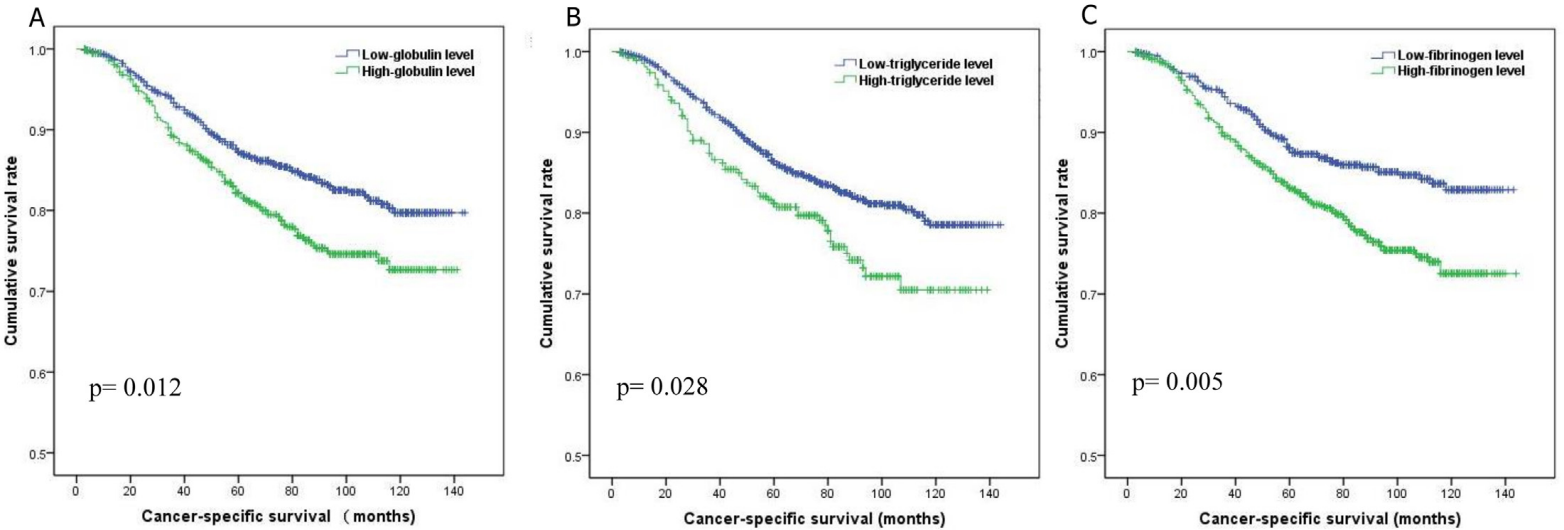
Cancer-specific survival of patients according to globulin level (A), triglyceride level (B) and fibrinogen level (C).

ROC curve analysis was performed and the AUCs were shown in S1 Table. The AUCs for albumin, globulin, LDH, triglyceride and fibrinogen are all statistically significant (all *p* < 0.05), and potential prognostic effects of total bilirubin and uric acid were not proved (*p* = 0.311 and 0.326 respectively). The optimal cut-off points for each potential prognostic factors were set up with the highest Youden index. Enrolled patients were stratified into high- or low-level by various variables. In the multivariate analysis, globulin (>30.0g/L), triglyceride (>1.10mmol/L) and fibrinogen (>2.83g/L) were identified as independent prognostic factors for poor cancer-specific survival in breast cancer patients (all *p*< 0.05, Fig 1).

The preoperative globulin, triglyceride and fibrinogen identified as independent prognostic factors in multivariate analysis were used to establish the prognostic index model (PI). The criteria of prognostic index model were as follows: globulin level >30.0g/L, triglyceride level >1.10mmol/L and fibrinogen level >2.83g/L were allocated 1 point each; globulin level ≤30.0g/L, triglyceride level ≤1.10mmol/L and fibrinogen level ≤2.83g/L were allocated 0 point each. The total score ranging from 0 to 3 was categorized into three prognostic index risk groups defined as: low-risk group, 0 or 1 point; moderate-risk group, 2 point; high-risk group, 3 point. There were 1168 (70.3%) patients allocated to low-risk group, 410 (24.7%) allocated to moderate-risk group and 83(5.0%) patients were categorized as high-risk group.

**Fig 2:**
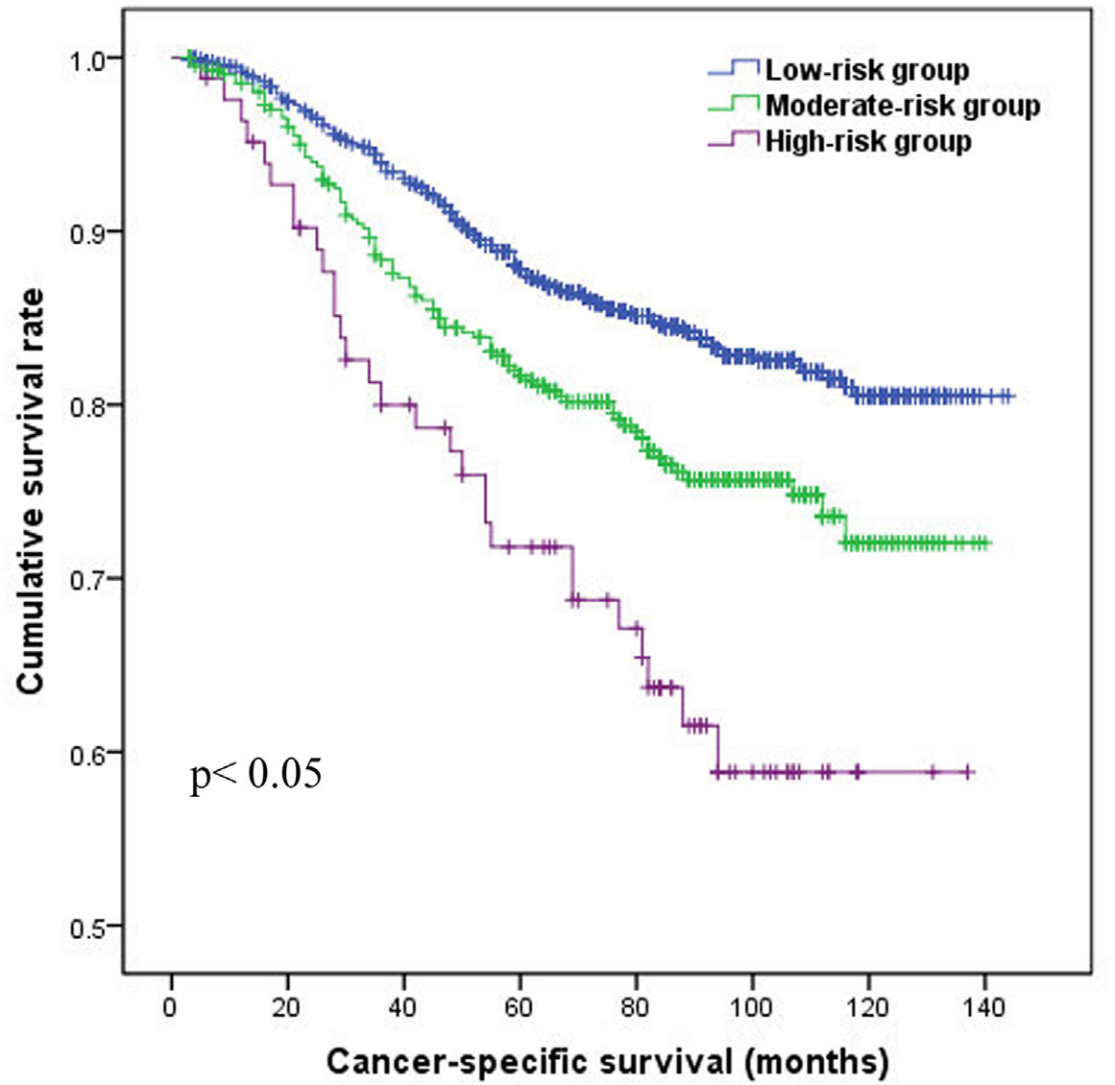
Cancer-specific survival of patients by prognostic index risk group.

The mean cancer-specific survival time for the low-risk group was 126.8 months (95% CI 124.5–129.2), which was significantly longer than that of 115.5 months (95% CI 111.1–112.0) in moderate-risk group and 99.7 months (95% CI 121.5–125.7) in high-risk group (both *p*< 0.05). The estimated 10-years survival rates for low-risk group, moderate-risk group and high-risk group were 80.5%, 72.0% and 58.8% respectively (Fig 2). Univariate analysis and multivariate analysis verified that the PI was significantly associated with CSS (HR = 1.513 for moderate-risk group and 2.481 for high-risk group, both *p*<0.05). In addition, menopausal status, tumor size, lymph node status, ER and HER2 status were identified as independent factors associated with CSS (all *p*< 0.05, Table 2).

**Table 2:** Univariate and multivariate analysis of PI for CSS in breast cancer.

Variable	Univariate analysis			multivariate analysis		
	Hazard ratio	95% CI	*p* value	Hazard ratio	95% CI	*p* value
Age	0.733	0.511–1.053	0.093	0.765	0.521–1.123	0.172
≤ 35	1 *(reference)*			1 *(reference)*		
> 35	0.733	0.511–1.053	0.093	0.765	0.521–1.123	0.172
Menopausal status						
Yes	1 *(reference)*			1 *(reference)*		
No	0.755	0.591–0.965	0.025	0.690	0.531–0.896	0.005
Tumor type	0.813	0.384–1.720	0.588	0.742	0.642–1.608	0.449
Histologic grade						
G1	1 *(reference)*			1 *(reference)*		
G2	0.711	0.536–0.943	0.018	0.781	0.522–1.170	0.231
G3	1.206	0.915–1.589	0.184	1.097	0.637–1.888	0.739
Tumor size						
T1	1 *(reference)*			1 *(reference)*		
T2	1.429	1.110–1.840	0.006	1.100	0.792–1.527	0.570
T3	3.830	2.620–5.601	<0.001	2.095	1.280–3.429	0.003
Lymph node status						
N0	1 *(reference)*			1 *(reference)*		
N1	2.043	1.462–2.854	<0.001	1.966	1.258–3.075	0.003
N2	3.646	2.600–5.111	<0.001	3.484	1.907–6.366	<0.001
N3	7.610	5.508–10.516	<0.001	6.080	3.410–10.842	<0.001
ER						
Negative	1 *(reference)*			1 *(reference)*		
Positive	0.577	0.460–0.725	<0.001	0.681	0.514–0.904	0.008
PR						
Negative	1 *(reference)*			1 *(reference)*		
Positive	0.612	0.485–0.773	<0.001	0.902	0.685–1.187	0.461
HER2						
Negative	1 *(reference)*			1 *(reference)*		
Positive	1.961	1.544–2.490	<0.001	1.335	1.018–1.751	0.036
PI						
Low-risk group	1*(reference)*			1*(reference)*		
Moderate-risk group	1.537	1.193–1.980	0.001	1.513	1.169–1.959	0.002
High-risk group	2.696	1.820–3.994	<0.001	2.481	1.653–3.724	<0.001

Abbreviation: CCS *Cancer-specific survival*, CI *confidence interval*, ER *Estrogen receptor*,PR *Progesterone receptor*, HER2 *Human epidermal growth factor receptor-2*, PI *prognostic index*

Under stratifications based on the molecular subtypes, the PI was independently associated with CSS in patients with Luminal A, Luminal B subtypes and triple-negative breast cancer (all *p*< 0.05, Table 3). However, no significant difference was observed in HER2 over-expressing among different PI groups.

**Table 3:** Multivariate analysis of PI and NPI based on various molecular subtypes.

Molecular subtypes	PI		NPI^2^	
	HR (95%CI)^1^	*p* value	HR (95%CI)^1^	*p* value
Luminal A	1.583 (1.135–2.207)	0.007	2.491 (1.859–3.338)	<0.001
Luminal B	1.885 (1.431–2.482)	<0.001	2.035 (1.557–2.660)	<0.001
HER2 over-expressing	1.352 (0.906–2.015)	0.139	1.260 (0.886–1.834)	0.227
Triple-negative breast cancer	1.579 (1.076–2.318)	0.020	1.631 (1.074–2.479)	0.022
All patients	1.570 (1.316–1.872)	<0.001	2.019 (1.724–2.365)	<0.001

^1^PI and NPI were processed as a continuous variable

^2^tumor size and lymph node status were in included in NPI multivariate analysis

Abbreviation: PI *prognostic index model*, NPI *Nottingham Prognostic Index*, HR *Hazard ratio* CI *confidence interval*, HER2 *human epidermal growth factor receptor-2*

As showed in Table 3, NPI was significantly predictive of CSS in all patients and in patients with Luminal A, Luminal B subtypes and triple-negative breast cancer, which was similar with the prognostic implication of PI. The prediction efficiency of PI was compared with that of NPI. The AUC of PI was 0.571 (95%CI:0.534–0.609) and that of NPI was 0.643 (95%CI:0.609–0.677). The difference between the two prognostic models was statistically significant (Z = 3.279, *p* = 0.001).

## Discussion

Breast cancer is the most commonly diagnosed cancers among women in the United States [1]. Despite newer therapies in the recent years, recurrence and metastasis remains the main challenge to the cancer management. About 30% to 40% of invasive breast cancer patients will eventually develop into metastatic breast cancer [20,21], who suffer low 5-year survival rate as 24%[22]. Generally, risk factors for poor prognosis include large tumor size, positive lymph node status, negative hormone receptor status and HER2 overexpression [23]. Moreover, previous studies have reported several factors related to poor outcomes of breast cancer patients, such as albumin, LDH [24], bilirubin and cholesterol [25].

In the present study, we retrospectively analyzed the preoperative parameters in breast cancer patients and identified globulin (>30.0g/L), triglyceride (>1.10mmol/L) and fibrinogen (>2.83g/L) as independent unfavourable prognostic factors for CSS.

The possible mechanisms of the above factors in tumorigenesis and progression were undefined. Globulins, generally including immunoglobulins and other acute-phase proteins such C-reactive protein (CRP), serum amyloid A, are the major component of serum proteins. An increased globulin level has been reported to reflect the presence of continuous systemic inflammation [26], which induces the increased levels of cytokines and promotes the tumor proliferation, progression, invasion and metastasis [27,28]. It was found that elevated alpha and gamma globulins were linked with poor prognosis in lung cancer and colorectal cancer patients [29,30]. Alpha-1 globulin could inhibit natural killer-cell activity and T cell-mediated cytotoxicity by suppressing cytotoxic reactions of lymphocytes [31]. Furthermore, COOH-terminal fragment, the degradation product of alpha-1 globulin by matrix metalloproteinase, could improve the tumor growth and invasion potential in vivo [32]. These biological behaviors regulate host anti-tumour defense mechanisms and promote tumor development [33]. Several studies have found a significant association between elevated serum triglyceride level and risk of breast cancer development [34,35]. Besides the active glycolysis, the lipogenesis is greatly increased in tumor cells [36]. The triglyceride/free fatty acid (TG/FFA) cycling plays an important role in multiple signaling pathways [37]. The TG/FFA cycling may promote cell survival through the activation of NF-κB and thus improve the expression of anti-apoptotic protein Bcl-2 and Bcl-x [38]. Hypoxic condition, which commonly occurred in the centre of solid tumors, could cause an elevation of triglyceride and expression of hypoxia inducible factor (HIF) that promotes metastasis of tumor [39,40]. The prognostic association between fibrinogen and cancer was reported by several studies. Fibrinogen can deposited around solid tumors and act as a stable framework to combine growth factors, such as fibroblast growth factor-2 (FGF-2) and vascular endothelial growth factor (VEGF), with tumor cells to increase the tumor proliferation and stimulate angiogenesis [14,41]. Moreover, fibrinogen can improve the adhesion of tumor cells to platelets, protecting tumor cells from the innate immune system and leading to an increase of metastatic cells [42].

Based on these three factors, we devised a Prognostic Index (PI) model and classified the enrolled patients into low-, moderate- and high-risk groups. Independently of the tumor size, lymph node status, hormonal receptor status and HER2 status, the PI was significantly associated with the overall postoperative survival, indicating that patients with higher PI experienced poorer prognosis.

Moreover, after stratification by molecular subtypes, preoperative PI remained significantly prognostic in Luminal A, Luminal B subtypes and triple-negative breast cancer. Generally, breast cancer patients with luminal subtype, especially Luminal A subtype, had relatively better clinical outcomes. Based on the preoperative PI, clinicians could identify patients with high risk of poor prognosis, and additional adjuvant treatment might be suggested beyond endocrine treatment. Moreover, both the PI and NPI could not effectively predict the prognosis in HER2 over-expressing subtype. That may be due to the carcinogenesis and the proliferation promotion effect of HER2 protein [43,44], which induce the unique characteristics of HER2 over-expressing breast cancer subtype.

The NPI combines three prognostic factors: nodal status, tumor size and histological grade, and the former two were found to be independently associated with survival in the present study. Patients with different NPI were considered to suffer distinguished prognosis. In the current study, the discriminatory ability of PI was compared with NPI, and the AUC value of NPI was significantly higher than that of PI (*p*< 0.05). Axillary lymph node status is the most important prognostic factor in the staging of the breast cancer patient [45,46]. The occurrence of axillary nodal metastases has been found to significantly decrease the 5-year survival rate up to 40%[47]. In the multivariate analysis, the hazard ratio of lymph nodal status was higher than that of PI, which may explain better discriminatory ability of NPI. Thus PI may act as complement to further improve the discriminatory ability of NPI.

The present study is limited to the retrospective nature. Firstly, selection bias cannot be excluded even though consecutive patients were included and eligibility criteria were performed to minimize the bias. Secondly, specific quality control analysis was performed by different quality inspectors since the laboratory parameters were obtained as routine clinical tests before surgery.

To sum up, our study identified three independent prognostic factors, globulin, triglyceride and fibrinogen, and set up a prognostic index (PI) based on these factors to stratify breast cancer patients into low, moderate and high risk of poor prognosis. These parameters are low-cost and routinely measured in clinical practice. Thus the PI may assist clinicians to identify high-risk patients and make personalized therapeutic approaches independently or along with other prognostic models.

## Supporting Information

S1 Table The optimal cut-off points and multivariate analyses of potential prognostic parameters. (DOC)

S2 Table Relevant data underlying the findings described in manuscript. (XLS)
