# Supplementary material for: The Practicability of a Novel Prognostic Index (PI) Model and Comparison with Nottingham Prognostic Index (NPI) in Stage I–III Breast Cancer Patients Undergoing Surgical Treatment
Source: PLoS One. 2015 Nov 23;10(11):e0143537. doi: 10.1371/journal.pone.0143537 (PMC4658156; doi:10.1371/journal.pone.0143537)
Supplement: S1 Table — (DOC) [file pone.0143537.s001.doc]

S1 Table. The optimal cut-off points and multivariate analysis of potential prognostic parameters

| Parameters | AUC | *P* value | Optimal cut-off points | Multivariate analysis | |
| --- | --- | --- | --- | --- | --- |
|  |  |  |  | HR (95%CI) | *P* value |
| Albumin | 0.457 | 0.021 | 49.0 g/L | 1.012(0.615-1.666) | 0.108 |
| Globulin | 0.547 | 0.014 | 30.0 g/L | 1.340(1.055-1.701) | 0.016 |
| LDH | 0.557 | 0.002 | 150.0 U/L | 1.093(0.850-1.405) | 0.489 |
| TB | 0.504 | 0.833 | - |  |  |
| Uric acid | 0.519 | 0.311 | - |  |  |
| Cholesterol | 0.518 | 0.326 | - |  |  |
| Triglyceride | 0.556 | 0.003 | 1.10 mmol/L | 1.384(1.036-1.850) | 0.028 |
| Fibrinogen | 0.585 | <0.001 | 2.83 g/L | 1.414(1.103-1.813) | 0.006 |

Abbreviation: AUC *area under the curves*, HR *hazard ratio,* CI *confidence interval,* LDH *lactate dehydrogenas*e, TB *total bilirubin*
